# Supplementary material for: Serum metabolome indicators of early childhood development in the Brazilian National Survey on Child Nutrition (ENANI-2019)
Source: eLife. 2025 Jan 15;14:e97982. doi: 10.7554/eLife.97982 (PMC11805503; doi:10.7554/eLife.97982)
Supplement: Supplementary file 1. — (a) Means and confidence intervals (CI) for developmental quotient (DQ) by sociodemographic variables of children 6–59 months evaluated in a subset sample of ENANI-2019 (the Brazilian National Survey on Child Nutrition) (n=5004). (b) Annotation of unknown ions from untargeted metabolome analysis of serum filtrate samples by MSI-CE-MS from the ENANI-2019 study. [file elife-97982-supp1.docx]

**Supplementary File 1:**

**Supplementary File 1a.** Means and confidence intervals (CI) for developmental quotient (DQ) by sociodemographic variables of children 6-59 months evaluated in a subset sample of ENANI-2019 (the Brazilian National Survey on Child Nutrition) (n = 5004).

| **Variables** | **Mean** | **95% CI** |
| --- | --- | --- |
| **Region** |  |  |
| North | 0.91 | 0.89; 0.93 |
| Midwest | 1.00 | 0.98; 1.02 |
| Southeast | 1.00 | 0.98; 1.02 |
| Northeast | 1.01 | 0.99; 1.04 |
| South | 1.02 | 0.99; 1.04 |
| **Age (months)** |  |  |
| 6 - 23 | 1.13 | 1.11; 1.15 |
| 24 - 35 | 0.99 | 0.97; 1.01 |
| 36 - 59 | 0.90 | 0.88; 0.91 |
| **Sex** |  |  |
| Male | 0.95 | 0.93; 0.96 |
| Female | 1.02 | 1.01; 1.04 |
| **Weight for length/height categories ^1,2^** |  |  |
| Underweight (z-score < -2) | 0.94 | 0.86; 1.03 |
| Normal (-2 ≤ z-score ≤ 1) | 0.98 | 0.97; 0.99 |
| Overweight risk (1 < z-score ≤ 2) | 1.01 | 0.98; 1.03 |
| Excessive weight (z-score > 2) | 1.00 | 0.97; 1.03 |
| **Maternal/caregiver education (years)** |  |  |
| 0 to 7 | 0.92 | 0.90; 0.94 |
| 8 to 10 | 0.98 | 0.96; 1.00 |
| ≥ 11 | 1.07 | 1.00; 1.03 |
| **Mode of delivery** |  |  |
| Vaginal | 0.98 | 0.97; 1.00 |
| Elective c-section | 0.98 | 0.96; 1.00 |
| Non-elective c-section | 0.99 | 0.97; 1.01 |
| **Monthly family income (USD) ^3^** |  |  |
| < 62.2 | 0.97 | 0.92; 1.02 |
| 62.2 - 124.4 | 0.92 | 0.88; 0.97 |
| 124.4 - 248.7 | 0.94 | 0.91; 0.96 |
| > 248.7 | 1.00 | 0.99; 1.01 |
| **Minimum dietary diversity (MDD) ^4^** |  |  |
| ≥ 5 food groups | 1.00 | 0.99; 1.01 |
| < 5 food groups | 0.96 | 0.95; 0.98 |

Note: CI: Confidence interval; USD: United States dollar.

^1^ 25 missing values; ^2^ Reference WHO child growth standards, 2006 (World Health Organization (WHO), 2006); ^3^ Estimated from the Brazilian minimum wage (R$ 998.00) and converted to the USD exchange rate (R$ 4.013 = USD 1) in 2019; ^4^ MDD: frequency of children who received ≥ 5 or < 5 out of eight food groups on the day before the interview, [food groups: (1) breast milk; (2) grains, roots and tubers; (3) beans, nuts and seeds; (4) dairy products; (5) flesh foods; (6) eggs; (7) vitamin A-rich fruits and vegetables; and (8) fruits and vegetables] (World and Health Organization, 2021) (World health Organization (WHO) & United Nations Children’s Fund (UNICEF), 2021).

**Supplementary File 1b.** Annotation of unknown ions from untargeted metabolome analysis of serum filtrate samples by MSI-CE-MS from the ENANI-2019 study.

| ***m/z*:RMT:mode^1^** | **Mean CV QC^2^ (%)** | **FD^2^ (%)** | **Blank Signal^2^** | **Putative ID^3^** | **HMDB#** |
| --- | --- | --- | --- | --- | --- |
| 104.0712:0.739:P | 16.1 | 75 | No | *Unknown cation*, from 8 viable cations & isomers^4^ | -- |
| 129.0660:0.634:P | 15.1 | 85 | No | Likely pyroglutamine, from 6 reported cations | 0062558 |
| 160.1338:0.594:P | 23.2 | 99 | No | Likely aminooctanoic acid, from 10 reported cations | 0000991 |
| 161.1281:0.410:P | 16.5 | 94 | No | Likely *N6*-methyllysine, from 6 reported cations | 0002038 |
| 276.1191:1.121:P | 17.5 | 83 | No | Likely *N*-*γ*-glutamylglutamine, from 6 reported cations | 0029147 |
| 334.6885:0.747:P | 24.3 | 72 | No | *Unknown* *cation*, no candidates reported | -- |
| 471.7380:0.865:P | 22.4 | 66 | No | *Unknown* *cation*, no candidates reported | -- |
| 117.0552:1.668:N | 20.8 | 94 | No | Likely 3-hydroxyvaleric acid, from 10 reported anions | 0000754 |
| 131.0344:0.817:N | 24.4 | 50 | No | Likely glutaric acid, from 7 reported anions | 0000661 |
| 135.0293:1.713:N | 13.2 | 88 | No | Likely erythronic acid, from 4 reported anions | 0000613 |
| 193.0348:1.303:N | 28.6 | 68 | No | Likely glucuronic acid, from 14 reported anions | 0000127 |
| 209.0297:3.038:N | 24.4 | 20 | No | Likely glucaric acid, from 2 reported anions | 0000663 |
| 274.1038:1.312:N | 18.3 | 47 | No | Likely *N*-*γ*-glutamylglutamine, same feature as cation 276.1191 | 0029147 |
| 313.4436:1.934:N | 15.3 | 93 | No | *Unknown* *anion*, no candidates reported | -- |

^1^ All unknown ions measured from serum filtrate samples were annotated based on their characteristic accurate mass (*m/z*), relative migration time (RMT) and ionization mode (positive, P or negative, N) when using MSI-CE-MS with full-scan data acquisition.

^2^ All unknown ions were also only reported provided that they were measurable with adequate precision in pooled QCs (CV < 30%) and consistency (frequency of detection, FD > 50%) without a signal measured in blank (No).

^3^ Unknown ions were tentatively identified from reported candidates in the Human Metabolome Database (HMDB) that were viable based on their charge state and reported concentration in blood (micromolar levels). In some cases, no candidates were reported on HMDB that coincided with their accurate mass for their protonated or deprotonated molecular ion with a mass error of 10 ppm.

^4^ Excluded isobaric cations that did not have consistent migration behavior included dimethylglycine, γ-aminobutyric acid and β-aminoisobutyric acid.
